# Guiding Preschool Play for Cultural Learning: Preschool Design as Cultural Niche Construction

**DOI:** 10.3389/fpsyg.2020.545846

**Published:** 2020-09-30

**Authors:** Robin Samuelsson

**Affiliations:** School of Culture and Education, Södertörn University, Huddinge, Sweden

**Keywords:** cultural learning, guided play, environment, play, cultural niche construction, cultural affordances, early childhood education, preschool

## Abstract

This paper explores how preschools can be purposefully designed to aid cultural learning through guided play practices. In recent literature, there has been a renowned interest in the role of the exogenous environment in psychological processes, including learning. The idea that the design of preschools can meaningfully be seen as cultural niche construction and that guided play practices in these environments can aid the preparation for cultural action is promoted, and a theoretical framework is presented. The empirical data draw from a synthesis from three ethnographic research sites in multilingual communities, and data are used to explore how cultural affordances are used in designed environments as part of guided play practices. The results indicate how niche construction of affordances aid cultural learning and is achieved through both direct guided play interaction between teachers and children and also in the way of the indirect design of environments that is incorporated in children’s peer play. It is discussed what this means for play research as well as for guided play practices that aim to promote cultural learning.

## Introduction

There is an ongoing debate on the role of play in early childhood. One area of concern is the decreasing time for children to engage in play during their childhood and in preschool settings (see Miller and Almon, 2009; Lukianoff and Haidt, 2018). Moreover, the debates also call into question the utility of play for pedagogical practices in early childhood. In an attempt to avoid too entrenched positioning in these matters, recent literature concerning *guided play* seems to have found a middle ground in between playful learning and academically oriented instruction for the preschool years (Toub et al., 2016).

These tensions have also been experienced in the Swedish context, where recent addendums promoting instruction in preschools have spawned debate, as they seemed to put tensions to the Swedish preschools’ renowned dual focus on education and care (“educare”), where play and playful learning formerly have been integrated to the preschool values and been a pillar of the curricula.

Play can, in one sense, be seen as emerging in the interaction between one, two, or several children. However, play is also in significant ways afforded by the preschool environment—in that the environment supplies children not only with the material boundaries but also with objects, toys, clothes, etc., for role-playing. Alongside this, recent literature in the behavioral sciences has suggested an increasing role of the cultural context and specifically its environmental affordances on cognition and learning (e.g., Clark, 2011). Weisberg et al. (2014) referred to this as the *mise-en-place* of learning, by promoting the particular role of the environment in playful learning encounters in the preschool age.

This paper takes a view of play and playful activity in preschool that aims to transcend the dichotomy of play vs. instruction, which has productively been addressed by the literature on guided play. Here, it is achieved through adding another dimension to the guided play model, by focusing on preschool design, and argues the notion that preschools provide a particular *cultural niche* that provides affordances, i.e., opportunities and constraints, on children’s learning through ways that can promote children’s own play or guided play practices at preschools.

Play is also the natural practice by which human children, other mammals, and even more distinct animal species engage in nonfunctional albeit often joyful and purposeful behavior (e.g., Burghardt, 2005). Moreover, play understood as an educational tool has been promoted by Hirsh-Pasek et al. (2009) as “guided play” or play that is initiated by children but in significant ways can be used by teachers to guide children toward cultural learning or other educational goals. Therefore, Zosh et al. (2018) have recently suggested a framework in *Frontiers in Psychology*, through which play can more productively be understood as a spectrum, ranging from free play to guided play to the more instructed type of games and activities. Play also provides a promising ground to study cultural learning, as Aronsson (2011, p. 464) noted how play builds on past play sessions and cultural events and thus “echoes” children’s past cultural experiences.

One side of guided play is that teachers themselves may enter into children’s play sessions to provide scaffolding interactions with the children, i.e., as in teachers playing together with children. Moreover, there is another side of guided play, in that teachers can intentionally design environments that aid children’s possibilities to learn from play. This paper suggests that there is an additional dimension to the spectrum that Zosh et al. (2018) suggested—in that the environment is often purposefully designed for cultural learning in preschool settings.

This study aims to provide evidence for how preschools can be arranged to support cultural learning through guided play. It probes into how teachers provide interaction and arrange environments in preschools that provide children with cultural affordances for play. This process is theoretically outlined to be a form of cultural niche construction. The paper empirically draws on data sets from three ethnographical projects (Ledin and Samuelsson, 2017; Samuelsson, 2018, 2020), conducted at low-socioeconomic status (SES) areas in Sweden where a majority of children are bilingual or multilingual. It is suggested that preschools can provide structured environments, with affordances that can deliberately be used to design settings that support learning for children emplaced in a new cultural and linguistic setting.

The paper sets out to examine the aim by focusing on two basic research questions:

1. How are the preschools designed to aid cultural learning through play?
2. In which ways does cultural niche construction affect play interactions among children and teachers?

To enable examination of these questions, we need to turn to research on how children’s play can be used as a pedagogical tool, i.e., research on guided play practices.

## Guided Play Practices in Preschool

For children in early childhood, play is a fundamental source of experience and learning. Educational programs in the preschool age that are based on play and playful instruction are, therefore, in the literature referred to as “developmentally appropriate” programs (Bjorklund and Causey, 2018). While it seems to be clear that play provides an essential ground for experience and learning in early childhood (e.g., Pellegrini, 2009), to what extent this is true is widely debated. Scholars such as Gray (2013) have argued that play is a more natural way of learning which can even be used past early childhood and is in a wide range of domains. Other play scholars, such as David C. Geary, take a more cautionary stance. Geary (2007, 2008) argued that while play provides a natural way for children to learn and develop, this is more limited to primary human faculties such as basic language learning and socialization. According to Geary, so-called secondary skills, usually associated with schooling, such as reading and mathematics, can only be learnt through repeated instruction.

Children’s free play without adult intervention indeed holds significant benefits for children (e.g., Bjorklund and Brown, 1998; Gray, 2015). Play may provide a range of adaptive benefits (see Burghardt, 2005), and even physical forms of play hold important benefits that might provide critical developmental benefits, not only to the development of physical movement but also to cognitive functioning (Pellegrini and Smith, 1998). While some of these benefits might be incidental, it should be underscored that due to the playful and unpredictable nature of play, it is cognitively demanding and hard, while also being underpinned by what Spinka et al. (2001, p. 142) called “the complex emotional state known as ‘having fun’.”

Recent research moreover shed light on the possible functions and benefits of play between adults and children. From neurocognitive perspectives, Piazza et al. (2020) provided evidence for “neural coupling” in synchronized activation patterns during play between an adult and a child. This synchronization is characterized by the interaction between the adult and child that is associated with communication and language, such as moments of joint attention and gaze, emotional coupling, and change in adult prosody (cf. Tomasello, 2019). These results become important when applied to guided play practices, as the interplay of the child and adult becomes critical.

Play-based programs in the preschool age provide the preschool child with a possible middle ground between instruction and play, or what Weisberg et al. (2013, p. 104) called *guided play*, in their review of evidence that suggests how play-based preschool programs can provide “a variety of positive academic outcomes” in later schooling. Guided play is conceptualized by Weisberg et al. (2016) as “learning experiences that combine the child-directed nature of free play with a focus on learning outcomes and adult mentorship […] Guided play thus has two key elements: child autonomy and adult guidance. This makes it engaging, but with the advantage of focusing the child on the dimensions of interest for a learning objective.” As play is an abundantly broad category of behaviors, it need not necessarily be categorized as providing “minimal guidance” (cf. Kirschner et al., 2006) for children. Play is not necessarily free or instructed; instead, in guided play, adults may provide ways of intentionally guiding or even instructing children toward learnable ends (Hirsh-Pasek et al., 2009).

Not only is guided play, or similar practices, shown to yield good results in longitudinal studies on academic outcomes (Stipek et al., 1995; Marcon, 1999), on a pedagogical and pragmatic level, guided play also dissolves age-old pedagogical debates surrounding the role of instruction in early childhood (e.g., Bonawitz et al., 2011), in the sense that guided play can allow learning that permits children exploratory behaviors, while also influencing children through teacher scaffolding, thus potentially retaining children’s engagement and joy often associated with play (Zosh et al., 2018). Importantly, the Weisberg et al. (2016) concept of guided play transcends some of the traditional barriers in pedagogical discussions that starkly contrast instructionally focused curricular approaches with others being deemed as play or discovery based.

In Sweden, it has been a tradition to schedule children to have time for “free play,” i.e., time for unobstructed peer play. Notably, this type of play is always within the preschool boundaries, and it thus becomes meaningful to conceptualize play in preschool as forms of guided play. Play can in this manner also be viewed as a spectrum encompassing everything from the unobstructed free play where children initiate and fulfill the play session themselves to various forms of guided or “co-opted” play where teachers instruct children in play and to playful learning activities that are both initiated and directed by adults (Zosh et al., 2018). From the angle of this paper, it will be illustrated how these spectra of activities are used to set the scene in the intentional design of preschool activities.

By utilizing the built environment in various ways, children can be promoted in their play around themes of interest. Nilsson (2009, p. 14) referred to this as a pedagogy of playworlds, defined as “joint participation of children and adults in a collectively created and shared world of fiction.” The pedagogy of playworlds has been influential in Scandinavian countries and builds on the work by Lindqvist (1995) and is implemented in enactments of children’s texts as carefully guided by an adult standing by the children. This is, in essence, seen as a creative endeavor by Lindqvist (2001), who emphasized the connection between children’s use of imagination in pretend play and creative enterprises such as art. The playworld approach to preschool pedagogy has been experimentally examined by Baumer et al. (2005), who showed significant results in children’s narrative development.

There are also other possible connections between children’s culture and play. Toub et al. (2018) used experimental conditions of book reading with children that were followed by free play, guided play, or a combination of direct instruction in play with artifacts relevant to the book. All children showed significant gains in vocabulary. However, the groups with adult interaction had higher scores, especially the group engaged through guided play.

The Swedish urban context of this paper is one defined by being set in a multicultural society, where monolingual children are in the minority in all of the preschools under study; in the preschool named Preschool 3, none of the children has Swedish as their mother tongue. This setting indeed affects how play is enacted within these settings. Cromdal (2001) noted frequently occurring code switching among bilingual children in play. Other studies have pointed out the multitude of communicative strategies among bilingual and multilingual children in play, where Björk-Willén (2007) observed imitational learning among children in play. Moreover, Ledin and Samuelsson (2017) observed how children used bodily communication to conduct play and that these encounters could be intercepted into guided play sessions by responsive teachers. The current study examines how learning possibilities for multilingual children are affected by how the niche of the preschool is designed by teachers.

## Theoretical Framework: Play as a Naturally Cultural Phenomenon

Humans are naturally affected by the culturally built and transferred environment, or the cultural niche (Flynn et al., 2013), in which one is emplaced. It is increasingly understood how human nature and human culture are bidirectionally influencing each other (e.g., Gottlieb, 2002; Lickliter and Honeycutt, 2003). Humans are distinguished in ways of building and transforming their environments to aid cultural transfer through social interaction. Flynn et al. (2013, p. 300) pointed out that cultural niches also have developmental properties in that environments are intentionally structured to facilitate the “rapid cognitive development and the acquisition of cultural information” from birth and onward. A salient feature of human culture is that it is arranged in what Sterelny (2012) called “learning environments,” which are niches intentionally set up to enhance cultural learning for children. This is true both for settings of formal schooling (Packer and Cole, 2019) and in different forms of learning through participation and apprenticeship (Rogoff, 1990; Lave and Wenger, 1991).

In psychological science, there has been a movement in theories of learning to incorporate features of the environment, such as cultural artifacts (Vygotsky, 1978) or extended means of reasoning (Clark, 2011) to whole distributed processes (Hutchins, 1995), as critical parts of the human cognitive functioning. Clark (2011) proposed that cultural niches often provide functions that enhance or extend our abilities to understand and solve problems in our cultural environments. Along these lines of reasoning, Weisberg et al. (2014) promoted the concept of a *mise-en-place* in processes of learning, discussing how the environment sets the stage for how learning processes can be managed. *Mise-en-place* entails how environments are being set up with certain resources, and Weisberg et al. (2014) turned this concept to the cognitive sciences to promote how cognitive processes also are staged in interaction with an environment.

The environment is also part of learning through communication, as Ochs and Schieffelin (2011, p. 10) adhered that “the built environment […] create communicative affordances.” Take the example of a traditional classroom where benches and their orientation afford a setup that directs children toward the lecturing by adults. In the paper’s context, a preschool designed for children’s participation (as in tables and utilities being built at child size and/or height) affords children’s play and participation. This will be evident in the following analysis and the provided examples of interaction, where the setting and its tools are actively incorporated in children’s play communication. In the paper, we turn to how preschools can be staged within a specific cultural and developmental niche to aid cultural learning through environments endorsing play.

In this sense, cultures and the interaction therein importantly provide children with guidance on how to act through what Ramstead et al. (2016) referred to as “cultural affordances.” These are cultural patterns that can be perceived by children and provide opportunities for cultural action when used. These can be seen in the behavioral routines, linguistic repertoires, and the socialization processes of the preschool (cf. Ochs and Schieffelin, 2011). Culture is typically transmitted in the language socialization that occurs through communication in a cultural setting. Importantly, this is an embodied practice that encompasses linguistic as well as whole behavioral routines adapted for the cultural niche. This process also intertwines language socialization with children’s play (Aronsson, 2011; Samuelsson, 2020). Here, it is hypothesized and explored that cultural affordances are used by children in play and that preschools are providing children with these through guided pedagogical practices.

Play is a fact of mammal species, hypothetically providing what Spinka et al. (2001) called “training for the unexpected.” Play also serves a particular cultural purpose for humans, not least in the preschool age (Bjorklund and Pellegrini, 2000). Vygotsky (1978) put forward the notion that play is in a fundamental sense built around cultural rules that children recruit onto their play and that, in play, children may act with “behavioral flexibility” (Pellegrini, 2009, p. 228) in regard to such rules, allowing children to try out cultural routines in play. Play can, in this sense, provide a practice ground for cultural engagement (Pellegrini, 2009), and in play, children can practice the limits of their developing understanding as social and cultural beings.

In our theoretical framework, cultural learning is seen as entwined with the affordances available in a particular cultural niche. In this view, the affordances of the environment are essential for cultural action. Thus, play in the culturally constructed niche of the preschool is providing an arena for *preparation for cultural action*, in the joyful and flexible manner that characterizes play.

Guided play is essentially describing the ways that adults, and specifically teachers, can involve themselves in children’s play to scaffold children toward learnable goals (Hirsh-Pasek et al., 2009). It can thus be suggested that guided play can hold potential for cultural learning, as adults can scaffold children in various ways in the, already, culturally infused play of children. Play, as studied in this paper, focuses on the use of cultural affordances, and as children’s play often is built around children’s accumulated cultural knowledge (Aronsson, 2011), it can also be seen as a proxy for cultural learning. Also, as play is essentially grounded in the acts of socialization that are provided by children’s participation in a culture (cf. Ochs and Schieffelin, 2011), it can be seen as an additional dimension to children’s socialization into a cultural niche and its language, routines, behavioral rules, etc.

### Theoretical Model: Cultural Niche Construction and Guided Play Practices

**FIGURE 1:**
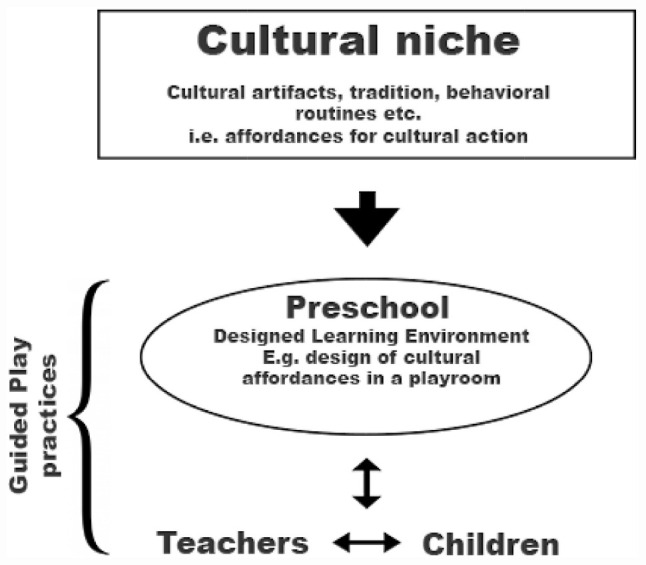
Visualization of the overarching theoretical framework describing cultural niche construction as integrated with guided play practices.

These levels of explanation are relevant in the guided play practices observed in the preschools of this study; to further explicate how this theoretical presupposition is understood, it has been further illustrated with the visualization in Figure 1.

In Figure 1, the theoretical model used in this paper is visualized. It is illustrated that preschools are constructed within a cultural niche and, as such, may draw on the different cultural affordances that might seem relevant when designing the preschool.

The research objectives of examining the preschool design and its affordances for children’s play thus become a part of the cultural niche. To enable the analysis, we turn to the multimodal methods that have been used to illuminate the embodied interaction of children, teachers, and the cultural environment.

## Materials and Methods

### Preschool Cases, Context, and Data Collection

The data are drawn from three ethnographic projects studying children in three departments at multilingual preschools (total *n* = 49). The ethnographic designs were influenced by child observational studies in natural settings (Pellegrini et al., 2012). The material gathered is multimodal in character, has been gathered from recordings in naturally occurring interactions at preschools, and has been collected with the intent to capture children play activity and both verbal and non-verbal behaviors in a rich context.

As far as data sets from three ethnographic projects are concerned, the paper has utilized a *qualitative synthesis* (Sandelowski and Barroso, 2006) of these projects. Data have thus been merged in the analysis for this paper to enable exploration of consistencies between the cases and providing some additional validity, despite the qualitative character of the study. Thus, the results presented regarding the characteristics of guided play can be seen as an aggregate of some findings from these three studied preschools. Also, sections with examples of local interaction from the three preschools are presented; here, a table is provided to give an overview of the collected data.

In Table 1, the amounts and types of relevant data collected during fieldwork at the preschools have been outlined. The character of the data can be clearly deemed as multimodal, primarily consisting of video/audio recordings but also of an array of photographs of environments, documentation, and activities. Different styles of interviews have been conducted, with the main objective of understanding the teachers’ intentions with their guided play practices and preschool environments. All data have been collected with a single device (audio or video) by the researcher, mostly arranged so that synchronized field notes can be taken during recording.

**TABLE 1:** Table overviewing data collected during the different projects.

Preschool	1	2	3	Summarization
Days of observation	3 days (shadowing two focal children)	7 days	11 days	
Primary data	5 h of audio recordings of activities with synchronous notes; field-notes during the days	Around 7 h of relevant video recordings	Around 11 h of relevant video recordings	23 h of recorded data (18 video, 5 audio/behavioral notes)
Supplementary data	Photos of environments; Informal interviews	Field notes; ∼100 photos of environments, artifacts, activities etc.; Informal interviews with teachers	Field notes, ∼250 photos of environments, artifacts, activities etc.; Semi-structured interviews with teachers	Around 360 photos
Age of children	3–5 years (group); Focal children 3 and 4	3–5 years	2–3 years	
No. of children	19 total (2 focal children appearing in almost all of the recorded data)	21	9	*n* = 49 total children
No. educators	3	3	2	*n* = 8

The data set from Preschool 1 followed two newly arrived focal children during subsequent days of a week. The dataset from Preschool 2 followed a preschool department for 7 weeks, conducted at weekly visits. The case of Preschool 3 followed a department of children in a multicultural area that has adopted a form of multicultural pedagogy.

The model of Swedish preschools has been called “educare” as it encompasses educational as well as caretaking activities in a holistic fashion (Jönsson et al., 2012). A typical preschool organization is seen in the data, in that approximately a third of the day consists of routines such as mealtimes and caretaking activities, a third of children’s time for play of their choice, and a third of planned activities such as more instructional circle time, projects, art activities, or visits in nature.

Any type of instructional activity is usually playful in character and almost never has the organization of a classroom instructional activity seen in school. This, and the substantial allotted time for children’s own play, almost necessitates teachers to use guided play practices to be able to scaffold children toward the goals of the curricula.

### Coding, Classification, and Analysis

As the data have been coded by the researcher, the procedures warrant detailed explanation. The data were preliminarily coded in field notes and later more thoroughly in NVivo according to children’s activities. Here, play activities were selected for further analysis by using the theoretical framework where play is seen as activities under children volition, often joyfully and with heightened interest that allows children to act with flexibility to the behavioral and cultural rules involved (see Burghardt, 2005; Hirsh-Pasek et al., 2009). This comprehensive definition captured both traditional play activities and activities that can be defined as guided or co-opted play (cf. Zosh et al., 2018). Play is notoriously hard to operationalize, and these theoretical definitions of play risk are too far from the empirical data. As such, a short excerpt from Preschool 1 is provided here as it clearly shows an activity that goes from being play to turning into nonplay.

**FIGURE 2:**
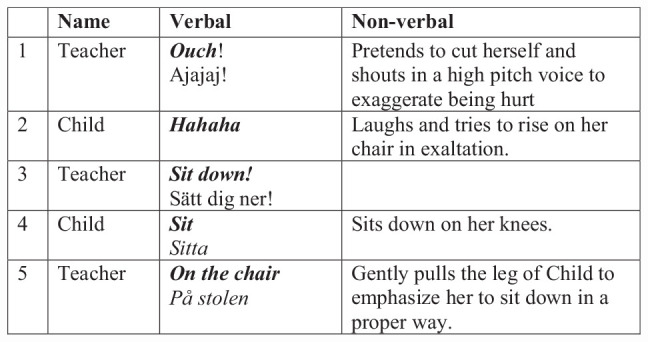
Teacher and child switch from joking to seriousness.

In Figure 2, an interaction around having fruit has turned into playfulness between a child and her teacher. The teacher pretends to cut herself in a joking way, but when the child gets overly excited by this, the pretend play cannot go on, and the teacher has to turn serious. In play, the interactants can flexibly pretend what may happen in the situation, but as it turns into nonplay, this flexibility disappears, as well as the joyfulness and child initiative. In other words, in play, the cultural affordances of mealtimes can be used in a flexible and playful manner, and we here see the cultural norms of sitting properly at a table being enforced as this flexibility is gone.

Through coding, two rough categories of teacher and preschool involvement in the children’s play were found; these were termed *direct guided play* and *indirect guided play*. These are simply defined as play where teachers directly interact face-to-face with children and when teachers influence children indirectly through their design of the environment. These categories were used as a guide for the analysis and write-up of the paper’s results.

After coding and extracting play activities from the data, a more systematic analysis could commence. Other aspects of this project have been published in Ledin and Samuelsson (2017) and Samuelsson (2019, 2020). Here, however, the notion of cultural affordances in guided play was taken as a central unit of analysis as it provides an intermediary between resources of the cultural niche, preschool design, and the behavioral flexibility provided by play behavior.

These activities underwent further analysis with particular attention to how cultural affordances were used by children in interaction. The analysis has been greatly inspired by studies of multimodality (e.g., Goodwin, 2000; Streeck, 2009; Bezemer et al., 2016) to provide tools for interactional analysis that attends to embodied human interaction as well as how it is influenced by the environmental affordances. In the paper, transcriptional procedures adapted from Jordan and Henderson (1995) have been used to separate verbal and other embodied modes of interaction into brackets, to aid readability of the complex interactions under study.

The multimodal analysis highlights how verbal language, embodied communication, and environments interplay in children’s play and thus returns us to the research objectives of how the cultural niche and its affordances are used in the preschool design and how these are used in play interaction.

### Ethics

The data collections were conducted in 2013, 2015, and 2020 in regulation with the Swedish Research Council code of conduct regarding information to and consent from participants and parents/caretakers, confidentiality, and ethical use of research data. In addition to this, the participating children have continuously been talked with to ensure their well-being and ongoing consent as well as to promote children’s understanding of the study.

In the presentation of data, all types of symbols or markers that might entail the preschools, the location, or the identity of children have been removed, and images of children have been masked. All names of children that are presented in the paper are fictitious.

Written informed consent was obtained from the minors’ legal guardian/next of kin for the publication of any potentially identifiable images or data included in this article.

## Results

After the operational definition of play stated above is established as the child-initiated activity where children can joyfully act with behavioral flexibility (i.e., activities with the character of the first part of Figure 2 and not the latter), the data could be more closely analyzed for how cultural affordances are used in the multimodal play interactions. It showed how children can use cultural affordances, in play, in a flexible manner. Three interactional examples, one from each of the three case preschools, will be used to further illustrate this.

The results indicate that guided play activities at the preschools can be divided into two general categories—those where teachers actively participate in play with children and those where teachers passively participate through their arrangement of the designed environment of the preschool, here termed as direct and indirect guided play.

Teachers also use direct forms of guided play in settings that they themselves have arranged. However, in the interactional examples provided, it will also be illustrated what may happen when children play in the designed preschool environments where cultural affordances are carefully provided as the teachers may indirectly guide children’s play through the cultural environment they arrange. Moreover, it is pinpointed how the indirect form of guided play can structure the interaction and emergent language use for bilingual and multilingual children as they enter into a new cultural niche, its language, and its cultural codes.

**FIGURE 3:**
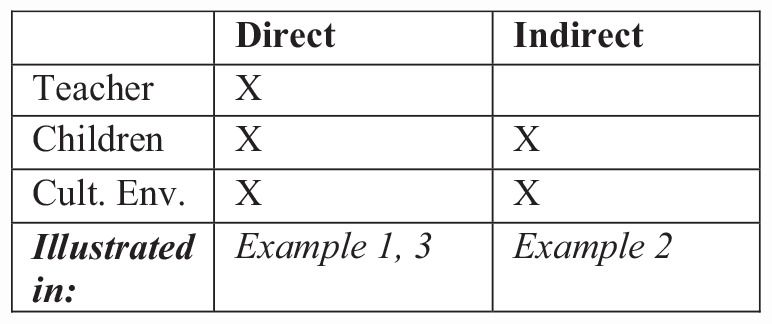
Mapping of types of Guided Play activities and their respective examples in the paper.

The overview of activities and the presence of teachers, children, and the active involvement of the environment and its cultural affordances are stated in Figure 3. In Figure 3, it is simply divided into how teachers, children, and environments are involved in the forms of guided play. It is moreover stated which of the interactional examples provided best illustrates these forms.

These types will be exemplified with excerpts from the interactional analysis to show how cultural affordances are at work in each of these situational types. Three examples follow in the next sections, providing one example from each of the three preschools.

### Preschool 1: Direct Use of Cultural Affordances in Guided Play Interaction

Preschool 1 features a large playroom where teachers can readily observe most of the children in the department and their activities or, when needed, join children to scaffold them in guided play, as shall be seen. The room featured in the following example is a smaller playroom with a store and household area, designed for children’s role-play that is connected with the larger playroom.

This example from Preschool 1 is from a guided play session where a teacher (Teacher 1) has entered into the play in a household play area at a preschool where two newly arrived children are playing: Li Na (4;0 years, Mandarin L1) and Dejan (3;9 years, Serbian L1). The children have started playing what seems like “fika,” the Swedish daily ritual of having coffee together. Since their play insofar has been mostly tacit, Teacher 1 has been engaging the children in verbal interaction around the theme of having coffee.

The play area presents some key items typical for a household scene: a child-sized stovetop, a table with small chairs, cutlery, plates, and toy food items. This excerpt is showing when another teacher (Teacher 2) enters into the room while Teacher 1 is sitting at the table and is ready to be pretendedly served “fika” by the children.

**FIGURE 4:**
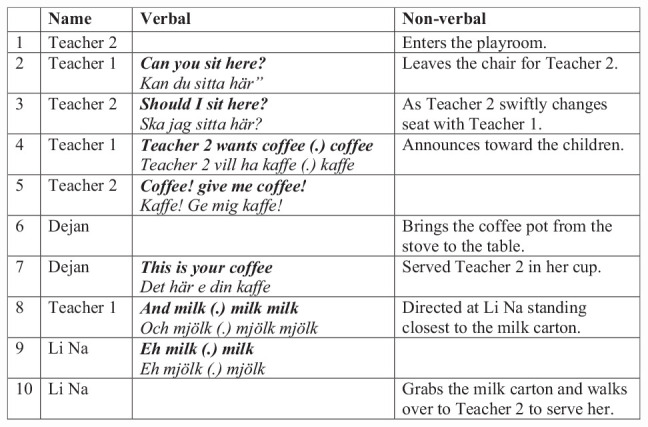
Teachers are guiding playing “having coffee.”

In Figure 4, Teacher 2 is immediately invited into the play by trading seats with Teacher 1 and engaged into the play session—“Teacher 2 wants coffee” or “give me coffee”; Teacher 2 promptly joins in (Lines 1–5). The play then turns into an informal language learning session, where coffee and milk are requested from the children, and corresponding items are brought to and pretendedly served to the teachers (Lines 6–10). Notably, the children manage this both by grabbing the correct items from the stove and table (coffee and milk) and, moreover, by imitating the words verbally.

The session shows how cultural affordances can be used in play. Here, a cultural scene is enacted that is typical in Sweden, where daily “fika,” i.e., sitting at a table and having coffee together, here is used as an informal language learning game for these children. The main structure is built around having a hot drink together and can, indeed, be seen across cultures and here seems to provide a familiar scene that can playfully be interacted around. The language used here seeps into play as an informal language learning session and thus provides a way for playful language socialization (cf. Ochs and Schieffelin, 2011) into the cultural niche. Language and other behavioral cues associated with “fika” are here afforded for the children as part of teachers scaffolding interaction, as well as by the environmental affordances.

Typical affordances are brought into interaction by the items that are present for the children, i.e., the coffee pot and milk carton. The items are, however, a part of the prop and design of this room, where emptied cartons or toy replicas of kitchen items are part of this playroom. These items afford the teachers and children to act out a culturally functional scene in their play. Notably, these are items that also featured in regular cultural activity but are more manageable for the children, i.e., the table is of a height appropriated for children’s play participation as well as the play stove without hot hobs. Thus, the example shows how cultural affordances can be playfully appropriated for children in guided play with the teachers, in an environment that is intentionally adapted for cultural learning in themes surrounding this everyday activity of having coffee together.

To further illustrate how the cultural niche of the preschool supports play on cultural themes, we next turn to children’s own peer play in a similarly culturally infused arranged preschool environment.

### Preschool 2: Indirect Use of Guided Play by Designing Environments

The second preschool is designed with several rooms that allow teachers to either have more instructional-type activities with a small group of children or as in the example taken here or allow children to play uninterrupted for longer bouts (some of the recorded sessions can last up to almost 1 h without teacher interference).

**FIGURE 5:**
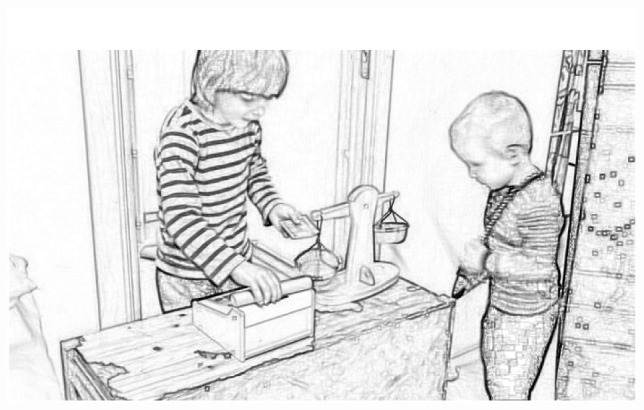
Children are playing store. One child has handed over a cash replica.

This excerpt is from the session pictured in Figure 5. Two children, Simon (4;1 years, English L1, Swedish L2, Tagalog L3) and David (4;11 years, Swedish L1), are playing store in an area of the preschool that is designed for this with the pictured cash register, replica bills, and items in the shop, ready to be bought.

**FIGURE 6:**
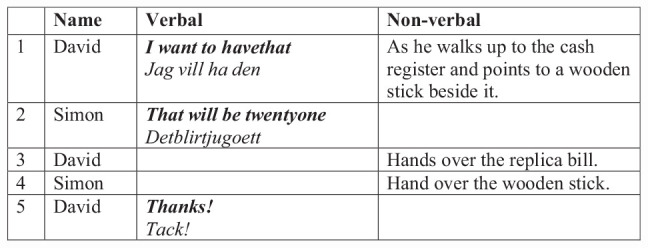
Children are playing a store transaction.

In this excerpt in Figure 6, David goes shopping in the store where Simon plays cashier. David here comes with a replica bill and requests an item from the store—“I want to have that” (Line 1). Simon then tells a price of 21 (Line 2), David hands over the replica bill (Line 3), and Simon hands over the wooden stick (Line 4). David thanks Simon for the exchange and leaves the store (Line 5).

Albeit being apparently simple, the children in this session enact what could be a functional transactional exchange at most stores. One of the children is playing in his second language; this, however, does not seem to present any barrier here, as the two children have played this type of scene before (both as reported by teachers and as seen in the observational data). The playroom also significantly structures this for the children by supplying them with the items needed to complete a store transaction. Notably, with only a few formulaic phrases and tacit knowledge of how to behave at a store, the children can play this functional store transaction, allowing children with an emerging second language to participate. Here, the power of design through cultural affordances, common to the present niche, can be seen. Children may iteratively reenact the cultural routine of a store transaction any number of times. Through this, they joyously practice the cultural behavior as well as common phrases used in this type of cultural scene.

The environment here provides a way for children to practice a store encounter; this is importantly aided by the cultural artifacts that are available for children to use in their play. Moreover, the environmental affordances can also be seen to steer children so that children are promoted to stand behind the counter, walk up to the counter, stand in line beside the counter, etc. Thus, this can be seen as an example of the *mise-en-place* (Weisberg et al., 2014), where the stage has been set up and propped for children’s successful play on themes of the store. Moreover, this exemplifies a general theoretical claim of this paper in that pedagogical play in preschool is in *preparation for cultural action*.

We now turn to how artifacts of culture themselves, namely, a children’s book, can be used in the preschool design and aid language learning in a multicultural community.

### Preschool 3: Using Cultural Affordances in and Out of Pedagogical Practices

The third section focuses on an ethnographic project in a thoroughly multicultural neighborhood. At this preschool, none of the children speaks the majority language at home, and only two of the children (*n* = 9) of the studied department share their first language. This fact is, however, thoroughly recognized and integrated with the preschool’s values and is reflected in pedagogical practice, as the preschool has designed several of the playrooms to aid multilingual play, such as having books in several languages and words with translations on the walls. This preschool’s multicultural practice is present in how teachers engage with the various linguistic backgrounds of the children and their families. The two teachers and their assistant speak a total of eight languages and are engaged with the children’s families and their home language and settings.

The preschool department studied, in this case, devotes a part of its practice to a children’s book that is available in most of the languages that are represented in the children’s homes. In this way, the work with the book can be continued in home settings—either by the preschool purchasing and supplying it or by parents being advised to visit the local library where it is available. In this way, the preschools’ practices can be continued outside of the confines of the preschool, not only through children’s play but also as a way of involving the community. The preschool has a dedicated involvement with parents and their various ethnic and linguistic backgrounds. As part of this, children’s books are made available in several of the first languages of children, and a local theater group is recruited to show a play of the book for the children.

**FIGURE 7:**
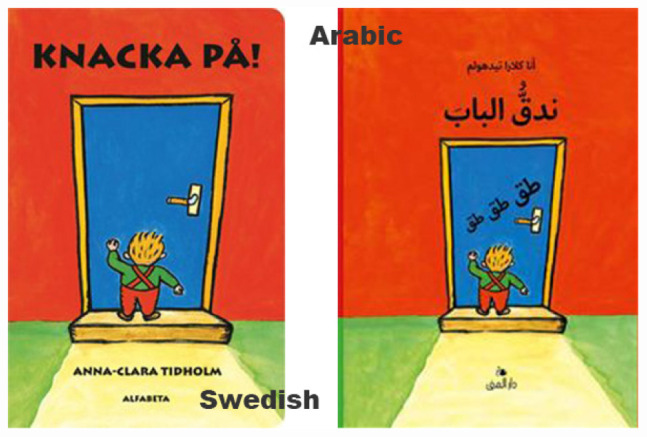
Cover of the book *Knacka på!* in Swedish and Arabic.

In Figure 7, a popular children’s book called *Knacka På* (*Knock, Knock, Knock!*) is shown in both Swedish and Arabic. The book features a house where different characters populate rooms of doors with a certain color. As part of the storytelling, children are advised to “knock on the blue/red/green/white door,” to interact with the book and the pages that feature colored doors.

The preschool teachers have chosen this book to be used as the theme of a project, where it has been implemented into the design of the preschool. The department under study has dedicated a small room to become the room inside the blue door from the book, shown in Figure 7. A cardboard door is made blue, and the teachers add various items featured in this room from the book, to make the room into a material reenactment of this room from the book.

With this design of the preschool environment, the teachers have created a space where children can play around items from the book, or teachers themselves readily can use guided play interactions to further activities related to the book.

**FIGURE 8:**
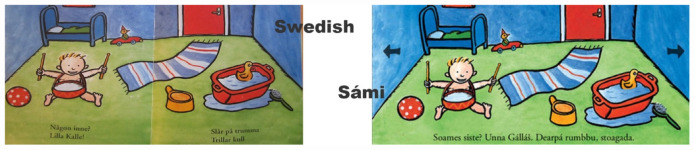
First room, as entered through the blue door, in Swedish and Sámi language.

**FIGURE 9:**
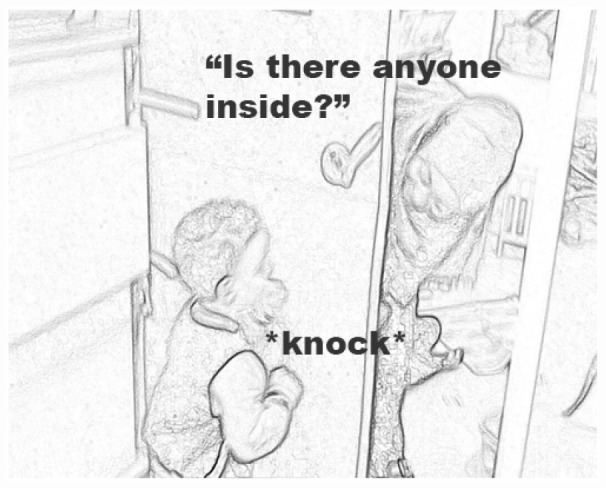
A child is invited by the teacher to knock on cardboard door.

In the following example, a teacher is engaging children to play inside the room. She has observed that a girl, Hana (2;6 years, Sorani L1), is playing with a duck in a small tub, as seen in the book (Figure 8). The girl’s solitary play engages the teacher who sets out to recruit children to play inside the room. In Figure 9, a key moment of interaction is shown. In the scene, a boy, Amir (2;5 years, Arabic L1), follow up on the teachers’ invitation to knock on the door and enter the playroom.

**FIGURE 10:**
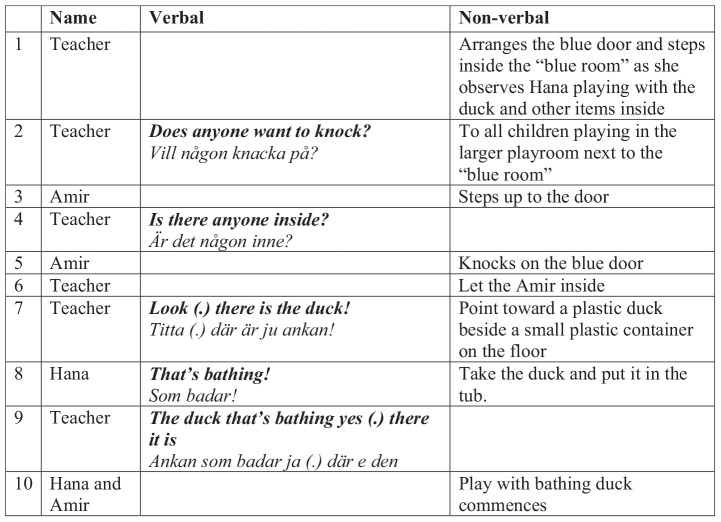
Teacher inviting the child to play inside the blue playroom.

In Figure 10, on Line 1, the teacher prepares the cardboard door and steps inside the room and then calls out into the next room to see if any child wants to join through using a phrase from the book—“does anyone want to knock?” The boy Amir, standing near the room, immediately shows interest and steps up to the door (Line 3). Amir is seemingly a bit unsure on how to follow up on this invite, which prompts the teacher to use another phrase from the book—“is there anyone inside?” (Line 4), and Amir follows with a knock on the cardboard door (Line 5).

As the boy is let inside the room (Line 6), the teacher exclaims toward the girl Hana, who is playing with the duck in the small tub, “look (.) there is the duck!” (Line 7). Hana responds by filling out the teachers’ sentence, “that’s bathing!” as she starts imaginatively bathing the duck in the tub (Line 8). The teacher confirmatively describes this, “The duck that’s bathing yes (.) there it is” (Line 9), and the children start to play with the duck and later dolls nearby.

The teacher can here spawn play with the children, and what the setup with the environmental design of the blue room affords the interaction is that she more readily can interpret the children’s intentions in play. As the teachers themselves have created this room and filled it with toys and items, it is readily available for play based on features of the themes in the book. Consequently, when the girl is playing on her own with a yellow duck and the design has thus provided affordances for play on this theme, it can easily be interpreted by the teachers and opens the readiness for guided play interaction. The design of this room is strikingly a case of *mise-en-place* (cf. Weisberg et al., 2014), as the room from the book is, quite literally, staged for children to play in.

This design of the playroom also makes the scaffolding process through guided play more predictable, as a teacher can more readily understand what children may play with in this room since they know what themes and items are featured in this room. The children are continuously observed to engage with the play themes from the book spontaneously, and parents have been informed over email and in person by the teachers about the book and were provided a word list with common items featured in the book. Moreover, parents are also advised to borrow the book and to make it available at the local library in several languages. The cultural affordances provided by the book are thus not only provided at the preschool or by happenstance at other times in the children lives but are intentionally integrated into the preschool design and supplied to families and the local community.

In the previous examples from Preschools 1 and 2, cultural affordances have been taken from common cultural practices (having coffee and going to store). In the example of Preschool 3, teachers have through the design of an environment provided children with affordances for children to engage with the linguistic and cultural repertoires featured in the book.

Cultural affordances may here provide a common ground for children’s play and for adults in this particular community to jointly enact themes from the book. Here, it has been adapted to fit with this multilingual community in a pedagogical practice that crosses boundaries of linguistic backgrounds. The children are observed to enact themes from the book (ducks, monkeys, and the child of the blue room named “little Kalle”) throughout the preschool day, and the preschool’s provision of cultural affordances here underpins a structure for children to do so.

## Discussion

This paper has examined various ways that teachers can guide or influence children’s play. It is forwarded that this is not only a matter of teachers’ direct interaction in guided play (cf. Zosh et al., 2018) but that this importantly holds a cultural dimension. It is proposed that the preparation of the cultural niche through affordances at the preschool can guide cultural learning through children’s play. This is basically provided by the feature of play allowing children to flexibly engage with cultural affordances, as children in play can practice an emerging cultural and linguistic understanding.

An important aggregated result from the analyses is how the local conditions at the preschool are reflected in the design of the preschool environment. A preschool such as Preschool 1, allowing teachers to overview children’s activity, can afford spontaneous guided play where teachers can scaffold children’s learning. In Preschool 2, children have more time for uninterrupted peer play. Preschool 3 is designed so that the children and their parents can be supported in learning the majority language.

In the direct form of guided play, teachers can enhance interaction through direct scaffolding of language and children’s behaviors. While this is not possible in indirect guided play, children can still incorporate earlier scaffolding by teachers. As Aronsson (2011) also described, children’s play builds on previous play and cultural experiences. This is evident where teachers have intentionally prepared the preschool environment in the indirect ways of guiding play described in this paper, and the results highlight how the environment should be seen as an important support in the pedagogical interactions and as affecting the preschool practices at large.

Play is seen as emplaced within a cultural niche, and this niche holds important developmental affordances for children (cf. Flynn et al., 2013). The hypothesis being promoted here is that preschools engage in forms of cultural niche construction, and it is hypothesized here specifically how these promote play. This is shown through how affordances are realized in the design of preschool environments and through deliberate interaction. The two main ways of guided play have been roughly outlined in this paper as being through teachers’ direct interaction with children or through the indirect environmental design of cultural affordances. It has also been shown how these are importantly conflated, as teachers act within and use the design of the preschool in their own interaction with children or even extendedly use the cultural artifacts with parents and the community as described in the case of Preschool 3, where the cultural affordances of a children’s book act as a common ground in a multilingual community.

Guided play has importantly been shown to provide a pedagogical model that can both be built on adult guidance of children and keep children’s natural inquisitiveness (e.g., Weisberg et al., 2016). We see this exemplified in Example 1, where children playing a household theme are being joined by one, and later two, teachers that use the active play theme to create a session of cultural learning of having Swedish “fika,” or coffee together. This is fundamentally afforded by the room, with various artifacts being used to enact the cultural scene. Here, cultural learning can be promoted while containing the spirit often associated with play behavior such as children’s engagement and joy (e.g., Spinka et al., 2001). This provides a “practice ground” for emerging cultural learning of children. This is an important empirical insight from the preschools studied in this paper, as children can practice a new language and cultural behaviors in ways that combine children’s engagement, teachers scaffolding, and a supportive pedagogical environment.

Moreover, a valuable insight underscored by the results of this paper has been that preschools can be deliberately designed by teachers to pedagogically support children’s learning and development. This is in essence seen as a form of cultural niche construction (Flynn et al., 2013), in that a cultural setting is here being arranged to aid children’s own exploration of cultural themes. In the spirit of guided play (Toub et al., 2016), this goes beyond the debates in education contrasting instructional vs. discovery-based approaches (e.g., Bonawitz et al., 2011), as even children’s own play in a setting can be set up to promote cultural learning. It is suggested from the analyses that this is importantly achieved through the intentional use of cultural affordances. The cultural niche thus, in extension, importantly steers children toward what is culturally meaningful but also limits possible actions. We see in Example 2 how children are playing store in an environment that in essence mimics the design of a store; here, children are aided to play out a store transaction over a store counter and are observed to repeat this type of bout several times. This provides an important structure for multilingual children, allowing flexible practice of a growing cultural repertoire.

General theories of play have posed that play is, in a fundamental way, about preparedness, as a practice for potentially upcoming live situations (Spinka et al., 2001; Burghardt, 2005). While this indeed is a compelling rationale for the nature of play, for modern humans, it also follows an important cultural function (Pellegrini, 2009). In the paper, we see how this can be used in a pedagogical manner, i.e., that the children in Example 2 can enact the play of a store transaction numerous times–it can then be suggested that play for children importantly is about *preparedness for cultural action*.

This is a possibly powerful insight for pedagogues. In Example 3, it is shown how a preschool extracts the cultural affordances of a popular children’s book to make a playworld (cf. Lindqvist, 1995) for children. Here, the children can repeatedly play in the setting of the room of the book by their own command, or guided play sessions can be spawned by teachers’ initiatives, as in Example 3 when a girl is already playing in the theme and a boy is invited into the girls’ play by the teacher. Here, a whole range of the spectrum of guided play can be seen (cf. Zosh et al., 2018)—as teachers are both designing the conditions for children’s play and engaging themselves in direct guided play within them.

These results are not least important for bilingual and multilingual contexts, such as the ones under study in this paper. Guided play is shown to steer children toward cultural learning in both direct and indirect ways. In this way, children of various backgrounds can be simultaneously scaffolded in both language and cultural learning in environments that let children immerse themselves through playful engagement in a new cultural setting. In Preschool 1, it is shown how teachers direct guided play through the use of cultural affordances and that this provides a scaffold for children’s language learning. This practice can productively be used in the many cases of integration of newly arrived children that today are commonplace in many early childhood settings, not least in Europe. Potentially, teachers can practice with children in guided play sessions like the one described here, as long as it remains playful for the children. Later, children can enact the same practiced routines themselves, much like the store transaction exemplified from Preschool 2.

Example 3 shows a case where the conditions of book reading and thematic play, which were experimentally shown by Toub et al. (2018) to promote children language development, are set in action at a preschool. In the example, teachers are using guided play interactions to engage children with the vocabulary of the book, similar to Toub et al. (2018). Additionally, it is also shown how the cultural affordances of the book can be used in the involvement with families and the community. Parents are sent information about the book and a wordlist, and the teachers also engage the local library and a theater. Thus, what is observed at the preschool in Example 3 is how the cultural affordances used in guided play can be used to promote learning both in and out of preschool. Thus, the design of preschools through cultural affordances provides possibilities not only for the pedagogical encounter itself but also for gathering children, educators, and parents around cultural themes. Cultural affordances are not only used for the design of the preschool environment but are moreover made available in this multicultural community environment in the case of Preschool 3, where parents, local libraries, and the theater are engaged to provide an array of cultural affordances that are initially drawn from the popular children’s book.

The Scandinavian pedagogical model of playworlds (cf. Lindqvist, 1995) is seen throughout the papers’ examples, as settings are being created to promote children’s imaginative engagement with different sorts of cultural themes. As Nilsson (2009) rightly underscored, part of the point of playworlds is that they are a joint creation of adults and children. This joint interaction can in this paper be separated into the direct and indirect forms of guided play, as a playworld might be created within the direct interaction of teachers and children, or as an indirect interplay between teachers, the preschool environment, and the children’s playful engagement within that environment.

The practice of designing preschools to take advantage of resources in the cultural niche is an example of the developmental properties of cultural niches (e.g., Flynn et al., 2013). The ethnographic data of this paper underline how this is a dynamic process, where interactions at the preschool influence how the preschool learning environment is designed and vice versa. This is a fundamental insight of the view of guided play that is forwarded here, in that it is a developing bidirectional relationship between children, adults, and the cultural environment as being meaningfully constructed within a cultural niche.

The preschool play environment can thus be seen as a form of what Sterelny (2012) called learning environment. It can be added to the model of cultural learning proposed by Sterelny (2012): how the design of a particular cultural niche in the context of preschools can be used to promote cultural learning through play. This is a novel insight into how learning environments might function in conjunction with guided play practices and also adds an indirect interactional factor to Sterelny’s (2012) apprenticeship model, as children may continue their participation in their play practices. This is also seen in how teachers can enjoin children’s playful engagement with cultural affordances and use this to guide children’s play, toward cultural learning (e.g., Vygotsky, 1978) and other forms of linguistic and behavioral socialization (e.g., Ochs and Schieffelin, 2011).

Here, we can also step back to the theoretical foundation of play as being a simultaneously natural and cultural phenomenon, reliant on both the environmental structures of the cultural niche and children’s propensity to play. One can trace back to the biological underpinnings of play (Piazza et al., 2020) and its reliance on “the complex feeling of ‘having fun”’ (Spinka et al., 2001, p. 145). This can be seen as a sort of gauge for guided play as well. If these emotional states are carried over to guided play sessions, they hold an apparent advantage for teachers of young children.

One should, simultaneously, also be careful of how much adults can tap into children play for pedagogical purposes. There is a risk that if too much play becomes pedagogical, free play is restricted (see Miller and Almon, 2009; Lukianoff and Haidt, 2018), and the essential forms of physical play (Pellegrini and Smith, 1998) are traded for seemingly more verbal or restricted counterparts, as the more physical and unrestricted types of play also hold important developmental functions. Here, a balance should be advised, as an early childhood pedagogy should understand the importance of free play, guided play, and the times where instruction is in place (e.g., Geary, 2007). The promises of guided play are, however, tremendous, and this paper has pointed toward some opportunities for teachers to both actively and passively guide children toward cultural learning while retaining some of the positive characteristics of play behavior. These opportunities are ever more relevant as groups of children in educational systems become more linguistically heterogeneous. In such cases, guided play as cultural niche construction is a perspective that simultaneously provides children with structure while allowing playful flexibility that can sustain children’s engagement in cultural learning.

## Conclusion

This paper has forwarded and explored the idea that playful learning environments can enhance cultural learning though a local form of cultural niche construction. This is practically achieved through the environmental design of cultural affordances. In guided play practices, children can be scaffolded in the said environments to steer children toward cultural learning. In short, the view of guided play as cultural niche construction incorporates environmental affordances for children play and underscores the importance of preschool play as *preparation for cultural action*. The preschool environment can be arranged to aid these practices, and in this paper, it has been empirically demonstrated, as well as theoretically outlined how this can be done in both *direct* interaction and in the *indirect* environmental design.

The role of play for children, both in ontogeny and phylogeny, is a long-lasting topic of inquiry and debate. This paper takes the view of play in the context of pedagogical practices, and specifically as guided play. While the potentials of play for pedagogical practice is becoming more evident, one should also recognize the role of unadulterated peer play for children. An important point drawn from these results, is, however, that guided play need not impinge on children’s time for play, but the papers’ results point to areas of enjoinment between educational goals and the positive emotional states associated with play.

The study also underscores the view of play to be a simultaneous cultural and natural phenomena. Children naturally engage in exploratory and play behavior in the world in which they are emplaced. The worlds in which we lived are meaningfully enriched through cultural evolution and play should be considered in models of cultural learning.

The paper enjoins the renowned interest in the role of environments in psychological processes. Further research that incorporates complex models that include the dynamic interactions of children, teachers and environments can supply important new results that go beyond simple discussions of either instructional *or* play-based perspectives on learning in early childhood.

Moreover, as it becomes clear that environmental and cultural affordances are important parts in the shaping of pedagogical practice in preschool, it should be a reoccurring part of the pedagogical agenda that the physical and cultural design of preschools are part of the didactical considerations made by teachers and others that interact with children in early childhood settings.

## Data Availability Statement

The raw data supporting the conclusions of this article will be made available by the authors, without undue reservation.

## Ethics Statement

Research was conducted under Swedish Research Council ethical standards. As no physiological or psychological intervention was done, no ethical board approval was needed. More details on research ethics are included in the Methods section. Written informed consent to participate in this study was provided by the participants’ legal guardian/next of kin. Written, informed consent was obtained from the minor(s)’ legal guardian/next of kin for the publication of any potentially identifiable images or data included in this article.

## Author Contributions

The author confirms being the sole contributor of this work and has approved it for publication.

## Conflict of Interest

The author declares that the research was conducted in the absence of any commercial or financial relationships that could be construed as a potential conflict of interest.
